# The Chemical Structures, Neuroprotective Effects, Structure–Activity Relationships, and Applications of Flavonoids, Phenolic Acids, Terpenoids, and Sterols in Dandelion: A Review

**DOI:** 10.1002/fsn3.71943

**Published:** 2026-06-10

**Authors:** Hongye Li, Bingchan Qu, Jiasu Wu, Menghan Sun, Youlin Xue, Tiejing Li, Chang Tan, Shan Wang, Chong Ning, Chongting Guo

**Affiliations:** ^1^ College of Light Industry Liaoning University Shenyang People's Republic of China

**Keywords:** active ingredients, application, dandelion, neuroprotection, structure–activity relationship

## Abstract

Dandelion, a plant belonging to the Compositae family, originated in Europe and is now widely distributed across the Northern Hemisphere. It is rich in bioactive compounds, including flavonoids, phenolic acids, terpenoids, and sterols, and exhibits a range of pharmacological activities such as neuroprotective, antioxidant, anti‐inflammatory, antibacterial, hypoglycemic, and hepatoprotective effects. The neuroprotective effects of its active constituents are primarily mediated through the inhibition of inflammatory responses, reduction of oxidative stress, and prevention of neuronal damage. This article further examines the structure–activity relationships of these compounds in relation to their neuroprotective effects. The analysis indicates that the pharmacological activities of dandelion components are largely determined by their core skeletons, while functional groups—such as hydroxylation patterns, glycosylation, and specific unsaturated bonds—fine‐tune their activity. As a non‐toxic plant, dandelion has been widely used in food and pharmaceutical applications. Therefore, this review summarizes current research on the chemical structures, neuroprotective effects, structure–activity relationships, and applications of flavonoids, phenolic acids, terpenoids, and sterols derived from dandelion in the medical and food industries, with the aim of promoting its further development and utilization.

## Graphical abstract

This review systematically summarizes the chemical structures and structure–activity relationships of key bioactive compounds in dandelion, including flavonoids, phenolic acids, terpenoids, and sterols. It highlights their neuroprotective mechanisms against neuroinflammation and oxidative stress, while outlining their promising dual applications in the food and medicinal industries.

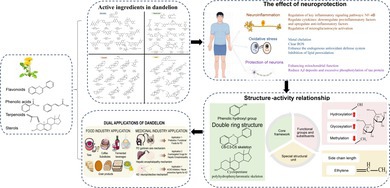

**Abbreviations**
AD: Alzheimer's disease
A*β*
: amyloid *β*‐protein
COX‐2: cyclooxygenase‐2
DES: Deep Eutectic Solvents
DF: dandelion flowers
DMB: dimethyl bisphenol
GCL: glutamate‐cysteine ligase
GPx: glutathione peroxidase
GSH: glutathione
H_2_O_2_
: hydrogen peroxide
IL‐10: interleukin 10
IL‐1*β*
: interleukin‐1*β*
IL‐4: Interleukin‐4
IL‐6: interleukin‐6
iNOS: inducible nitric oxide synthase
LPS: lipopolysaccharide
MAE: Microwave‐Assisted Extraction
MAPK: mitogen‐activated protein kinase
MDA: malondialdehyde
MLF: mulberry leaf flavonoid
NF‐*κ*B: nuclear factor kappa B
NO: nitric oxide
Nrf2: nuclear factor erythroid 2‐related factor 2
O_2_^−^
: superoxide anions
OH: hydroxyl radicals
OS: oxidative stress
PLE: Pressurized Liquid Extraction
RNS: reactive nitrogen species
ROS: reactive oxygen species
SFE: Supercritical Fluid Extraction
SOD: superoxide dismutase
TAX: taraxasterol
TLR4: Toll‐like receptors 4
TNF: tumor necrosis factor
TNF‐*α*
: tumor necrosis factor‐*α*
TOE: dandelion extract
UAE: Ultrasound‐Assisted Extraction

## 1 Introduction

Neurodegenerative diseases typically involve synaptic loss and neuronal death, leading to decreased cognitive and motor abilities (Labzin et al. 2018). Globally, neurodegenerative diseases such as Alzheimer's disease (AD) (Mary et al. 2024), Huntington's disease (Koriath et al. 2024), and Parkinson's disease (Chou et al. 2021) have become increasingly important issues affecting human health and quality of life. Effective treatments for neurodegenerative diseases are non‐existent, and clinical drugs such as selegiline and levodopa are limited to alleviating symptoms and exhibiting side effects. Therefore, developing novel, safe, and effective neuroprotective drugs and natural active ingredients has become a research hotspot in neuroscience. Natural products, particularly traditional herbs, are potential sources of neuroprotective drugs because of their rich biologically active components and a history of clinical applications.

Dandelion, plants of the genus Taraxacum, is a member of the Asteraceae perennial herbaceous plant family (Yao et al. 2022). It is a rich resource believed to have originated in Europe and is currently distributed throughout the Northern Hemisphere, including Northern Europe, the temperate zone of North America, and Asia (Lis and Olas 2019). In China, dandelion is widely distributed in the northern regions, including Gansu, Shanxi, and Xinjiang, is adaptable, and can grow in all types of soils from sea level to high altitudes (Garcia‐Oliveira et al. 2021; Grauso et al. 2019). Dandelion is a wild vegetable and traditional Chinese medicine that the Ministry of Health has classified as a medicine food homology (Qian et al. 2014). For example, its dried whole plant is used in traditional Chinese medicine for its neuroprotective, antioxidant, anti‐inflammatory, antibacterial, hypoglycemic, and hepatoprotective effect, as mentioned in Arabian, Indigenous American, Chinese, and Ayurvedic medicine. As dandelion is rich in nutrients, including flavonoids, phenolic acids, terpenes, and sterols, it is a nutritious food ingredient. The fresh leaves and flowers of dandelion can be consumed in raw and cooked forms. Recently, an increasing number of studies have demonstrated that the active ingredients in dandelion have a high potential for neuroprotective effects. Therefore, this paper reviews the research progress on the chemical structure, neuroprotective effects, structure–activity relationships, and applications in the fields of medicine and food of flavonoids, phenolic acids, terpenoids, and sterols in dandelion.

## 2 Active Ingredients in Dandelion

Dandelion is rich in diverse bioactive compounds, including flavonoids, phenolic acids, terpenoids, and sterols, which play significant roles in various biological functions. However, the genus *Taraxacum* is one of the most taxonomically complex angiosperm groups, comprising over 2500 microspecies distributed across temperate and subtropical regions worldwide (Tanasa et al. 2025). In addition to the most extensively studied species (*T. mongolicum*, *T. coreanum*, and *T. officinale* ), growing evidence indicates that other taxonomically distinct species—including *T. kok‐saghyz* (Russian dandelion, Central Asia), *T. platycarpum* (Korea, Japan), *T. formosanum* (Taiwan), *T. laevigatum* (Europe), *T. hondoense* and *T. obovatum* (Japan, Spain)—possess unique phytochemical profiles that translate into species‐specific neuroprotective potential (Fan et al. 2023). For example, an LC–MS/MS‐based comparative metabolomic analysis identified distinct chemotypes characterized by sesquiterpene lactones, phenolic inositols, and chlorogenic acid derivatives, highlighting that interspecific chemical heterogeneity is the rule rather than the exception (Lee et al. 2021). Therefore, this section extends beyond commonly cited species to include underexplored taxa, providing a more comprehensive overview of the genetic and chemical diversity underlying the neuroprotective potential of the *Taraxacum* genus.

### 2.1 Flavonoids

**FIGURE 1:**
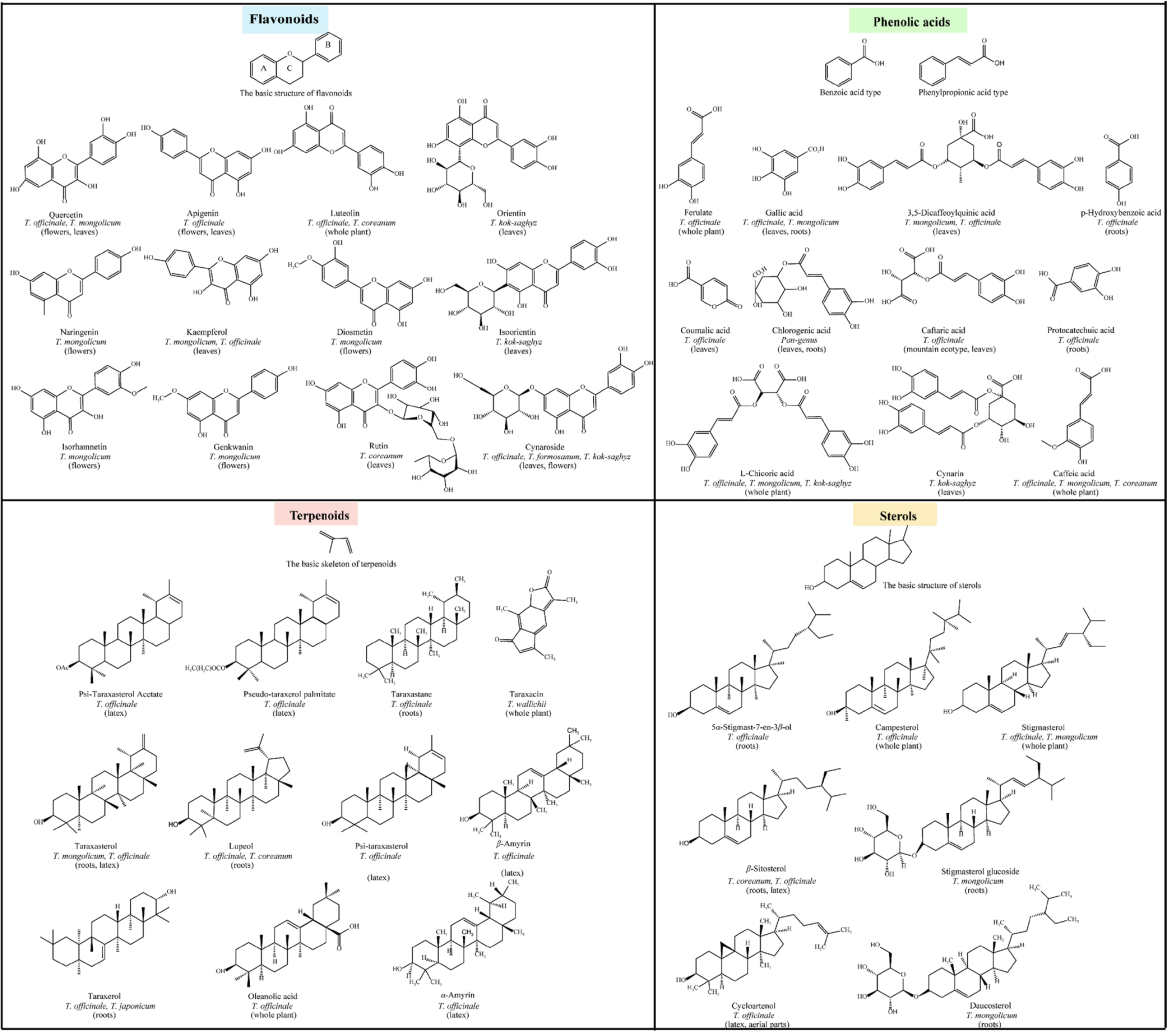
The chemical structures of dandelion flavonoids, phenolic acids, terpenoids, and sterols.

Flavonoids are water‐soluble compounds abundantly present in fruits, vegetables, and their derivatives, predominantly in the form of glycoside conjugates (Rocchetti et al. 2022). They are plant phenolic secondary metabolites and constitute the largest and most extensively researched class of plant metabolites to date (Munteanu et al. 2016). The primary structure of these compounds comprises three rings: two aromatic rings (A and B) and a heterocyclic pyran ring (C), adopting a C6‐C3‐C6 configuration (Qi et al. 2023). Flavonoids are essential active components in dandelion, making up approximately 1.35% of the entire herbaceous plant. Dandelion flavonoids can be categorized into various types, including isorhamnetin, kaempferol, apigenin, rutin, naringenin, cynaroside, luteolin, and quercetin (González‐Castejón et al. 2012), and their structures are illustrated in Figure 1. Notably, these compounds exhibit antitumor, antioxidant, anti‐aging, and immunomodulatory functional activities (Anand David et al. 2016).

### 2.2 Phenolic Acids

Phenolic acids are a class of metabolites containing phenolic hydroxyl and carboxyl groups, found widely throughout the plant kingdom. The main structural types of phenolic acids can be divided into two categories: the C6‐C1 type, which has benzoic acid as its core structure, such as gallic acid; and the C6‐C3 type, with phenylpropionic acid as its basic backbone, such as caffeic and ferulic acids (Li et al. 2024a). Phenolic acids in dandelion include gallic, chlorogenic, protocatechuic, l‐Chicoric, coumaric, p‐hydroxybenzoic, caffeic, and ferulic acids (Schutz et al. 2006), with their structures shown in Figure 1. Notably, these phenolic acids exhibit antiviral, anti‐inflammatory, antibacterial, immune‐boosting, antioxidant, and free radical‐scavenging properties (Xu et al. 2021).

### 2.3 Terpenoids

Terpenoids are a class of natural products derived from methyl hydroxypropanoic acid with the general formula (C_5_H_8_)_n_, along with various oxygen‐containing derivatives and compounds with different degrees of unsaturation. They are formed by the linkage of isoprene units in different ways. The major terpenoid components in dandelion are triterpenoids and sesquiterpenoids, primarily including psi‐taraxasterol acetate, pseudo‐taraxerol palmitate, taraxasterol (TAX), lupeol, taraxastane, psi‐taraxasterol, taraxerol, and oleanolic acid (Lin et al. 2019) (Figure 1). These compounds exhibit biological activities such as anti‐tumor, antibacterial, anti‐inflammatory, immune‐regulatory, and cholesterol‐lowering properties (Jiao et al. 2022).

### 2.4 Sterols

Steroid compounds, as some of the most important natural products, feature a perhydrocyclopentanophenanthrene as their core structure. The parent nucleus has four rings: three six‐membered and one five‐membered. The C3 position of the parent nucleus is substituted with a hydroxyl group, the C10 and C13 positions are substituted with methyl groups, and the C17 position has a side chain. Common dandelion sterol compounds include: 5*α*‐stigmast‐7‐en‐3*β*‐ol, campesterol, *β*‐sitosterol, stigmasterol, and stigmasterol glucoside (Fan et al. 2023) (Figure 1). These compounds exhibit various biological activities, including anti‐inflammatory, antioxidant, antiviral, anti‐cerebral ischemia/reperfusion injury, and antitumor effects (Qin et al. 2024; Ren et al. 2022; Yang et al. 2021).

### 2.5 Comparative Analysis of Extraction Strategies for Dandelion's Active Components

The extraction efficiency of neuroprotective constituents from dandelion is governed by the interplay of solvent polarity, mass transfer kinetics, and compound stability. A comparative evaluation of extraction techniques, optimal conditions, and yields is summarized in Table 1 (Simándi et al. 2002; Fan et al. 2023; Huber et al. 2015; Jia et al. 2022; Jiao et al. 2022; Lis et al. 2019; Liu et al. 2025; Liu et al. 2020; Milovanovic et al. 2024; Sun et al. 2014; Sutkaitiene et al. 2025; Wang et al. 2023; Yang et al. 2022; Yazici 2021; Yoon et al. 2025).

**TABLE 1:** Comparative assessment of extraction techniques, optimal conditions, and yields of active components from dandelions.

Target class	Marker compound	Plant species (source)	Extraction method	Conditions and solvents	Yield	Advantages and limitations	References
Flavonoids	Total flavonoids	*T. mongolicum*	Hot Ethanol (Reflux)	60% Ethanol, 80°C, 70 min	141.2 mg/g	High yield but high thermal degradation risk.	Wang et al. (2023)
		*T. mongolicum*	Solid‐State Fermentation	*Eurotium cristatum*	66.1 mg/g	Bio‐conversion enhances release; complex process.	Liu et al. (2020)
		*T. mongolicum*	UAE	39.6% Ethanol, 43.8 min	26.2 mg/g	Balanced yield and energy efficiency.	Sun et al. (2014)
		*T. mongolicum*	MAE	38.7% Ethanol, 2.86 min	24.1 mg/g	Fastest method (< 3 min); slightly lower yield than UAE.	Jia et al. (2022)
		*T. officinale*	SFE‐CO_2_	CO_2_	25.9 mg/g	Solvent‐free, eco‐friendly; high equipment cost.	Milovanovic et al. (2024)
		*T. assemanii*	UAE optimized by RSM	68% Ethanol, 46°C, 88 min, 1:37 g/mL	13.16 ± 0.91 mg GAE/g DW	Optimizes 4 variables for high yield with low energy/solvent and cavitation protection at 46°C, but requires complex pre‐experiments.	Yazici (2021)
Phenolic Acids	Chicoric acid	*T. officinale*	UAE	41.7% MeOH, 75°C, 51.8 min	26.09 mg/g	Best for heat‐sensitive esters; prevents hydrolysis.	Lis et al. (2019)
		*T. mongolicum*	UAE	55% Ethanol, 45 min	12.2 mg/g	Lower yield than optimized MeOH conditions.	Yang et al. (2022)
	Chlorogenic acid	*T. mongolicum*	DES‐UAE	Betaine‐Oxalic acid (DES)	4.27 mg/g	Green solvent; high selectivity for target acid.	Liu et al. (2025)
Terpenoids	TA‐G (Sesquiterpene)	*T. officinale*	Direct Maceration	Fresh Latex + Methanol	70 mg/g FW	Highest concentration source; avoids drying loss.	Huber et al. (2015)
	Taraxasterol	*T. mongolicum*	Solvent extraction	Ethanol/Methanol system	3.013 μg/mL	Low specificity; co‐extracts large amounts of matrix interferences; low purity.	Jiao et al. (2022)
	*β*‐Amyrin	*T. officinale*	SFE‐CO_2_	45 MPa, 65°C	0.045 mg/g (Dry weight)	High selectivity; eliminates polar impurities.	Simándi et al. (2002)
	Sesquiterpene lactones	*T. laevigatum, T. disseminatum*	Chromatography	Ethanol extraction	8 sesquiterpenoids isolated	Species‐specific lactone profiles	Fan et al. (2023)
Sterols	*β*‐Sitosterol	*T. coreanum*	PLE (Pressurized Liquid)	Ethanol, High Temp/Press	3.91 mg/g	Highest Yield; significantly outperforms Soxhlet.	Yoon et al. (2025)
		*T. coreanum*	Soxhlet	n‐Hexane (Reflux)	3.34 mg/g	Standard method; time‐consuming (6–24 h); toxic solvent.	Yoon et al. (2025)
		*T. officinale*	Traditional Reflux	Ethanol (Atmospheric)	0.07 mg/g	Inefficient; poor solubility of sterols in cold ethanol.	Sutkaitiene et al. (2025)

Abbreviations: DES, Deep Eutectic Solvents; FW, Fresh Weight; MAE, Microwave‐Assisted Extraction; PLE, Pressurized Liquid Extraction; SFE, Supercritical Fluid Extraction; UAE, Ultrasound‐Assisted Extraction.

#### 2.5.1 Hydrophilic Compounds (Phenolics & Flavonoids)

For polar bioactive compounds such as flavonoids and phenolic acids, preventing thermal degradation of target molecules is a primary challenge. While traditional hot ethanol reflux achieves the highest total flavonoid yield (141.2 mg/g), it poses a significant risk of degrading heat‐sensitive compounds. To address this limitation, modern techniques such as ultrasound‐assisted extraction (UAE) and microwave‐assisted extraction (MAE) are widely employed. MAE offers notable kinetic advantages, completing extraction in under 3 min (24.1 mg/g), whereas UAE operates under milder conditions and effectively prevents the hydrolysis of heat‐sensitive esters such as chicoric acid (26.09 mg/g). Furthermore, statistical optimization approaches, such as response surface methodology (RSM) applied to *T. assemanii*, can maximize extraction yields (13.16 mg GAE/g) at relatively low temperatures (46°C) while minimizing solvent and energy consumption. Emerging green strategies—including deep eutectic solvents (DES) for selective chlorogenic acid extraction (4.27 mg/g) and solid‐state fermentation for enhanced bio‐conversion—offer promising alternatives to conventional organic solvents.

#### 2.5.2 Lipophilic Compounds (Sterols & Terpenoids)

In contrast, the extraction of lipophilic constituents such as terpenoids and sterols is constrained by solubility and diffusion limitations. For sterol recovery (e.g., *β*‐sitosterol), pressurized liquid extraction (PLE) significantly outperforms conventional Soxhlet and atmospheric reflux methods, achieving yields of up to 3.91 mg/g by utilizing elevated temperature and pressure to reduce solvent viscosity and overcome matrix resistance. For terpenoids, supercritical fluid extraction with CO_2_ (SFE‐CO_2_) provides high selectivity. Although the absolute yield of specific triterpenes like *β*‐amyrin may be relatively low (0.045 mg/g), this method effectively eliminates polar impurities, making it suitable for obtaining highly enriched fractions. Targeted pretreatment strategies are also critical. For instance, direct maceration of fresh latex prevents the loss of high‐concentration sesquiterpenes (70 mg/g FW) during drying. Meanwhile, conventional ethanol extraction followed by advanced chromatographic techniques remains essential for capturing structural diversity, enabling the isolation of species‐specific sesquiterpene lactone profiles (e.g., 8 distinct sesquiterpenoids) from less‐studied species such as *T. laevigatum* and *T. disseminatum* .

Overall, current evidence supports a fractionated extraction approach: initial recovery of antioxidant phenolics using rapid MAE or optimized UAE, followed by PLE or SFE for the selective isolation of anti‐inflammatory sterols and terpenoids.

## 3 Neuroprotective Effects of Active Ingredients of Dandelion

When exploring the pathogenesis of neurodegenerative diseases, the relationship between neuroinflammation (Castro‐Gomez and Heneka 2024), oxidative stress (OS) (Liu, Chen, et al. 2017), and neurons (Huang et al. 2018) is particularly important.

**FIGURE 2:**
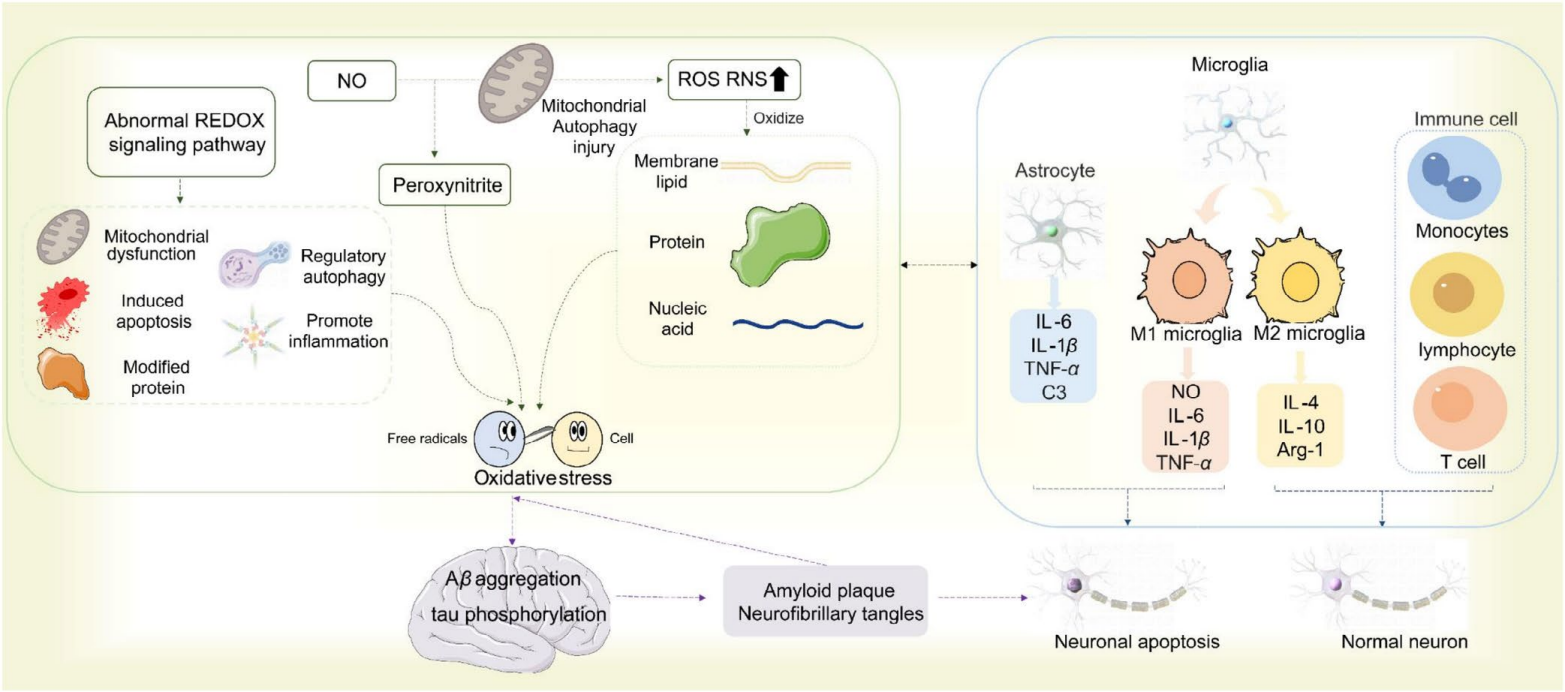
Mechanisms of inflammation, oxidative stress, and neuronal interactions.

Neuroinflammation causes chronic OS (Cassidy et al. 2020). Microglial activation can increase further OS and neuronal death through ROS and reactive nitrogen species (RNS) (Gu et al. 2021). OS is a major cause of neuronal cell death, due to the brain's rich fatty acid content and low antioxidant capacity (Li et al. 2021). OS also promotes the aggregation of amyloid *β*‐protein (A*β*) and the phosphorylation of tau protein, forming amyloid plaques and neurofibrillary tangles, which accelerates the death of neurons and exacerbates the vicious cycle between neuroinflammation and OS (Dash et al. 2024) (Figure 2).

The onset of neurodegenerative diseases involves complex interactions among neuroinflammation, oxidative stress, and neuronal damage, highlighting the need for protective strategies that can coordinately modulate multiple pathological processes. Plants of the genus *Taraxacum* are rich in bioactive components such as polyphenols, flavonoids, and terpenoids, which show the potential to simultaneously intervene in these processes, making them promising candidates for neuroprotection. Existing research on their mechanisms can be categorized into two approaches: first, direct evidence based on dandelion extracts, reflecting the holistic bioactivity of the plant as a complex system; and second, mechanistic evidence based on isolated standardized compounds (e.g., chicoric acid, luteolin, taraxasterol), which aims to elucidate their precise molecular targets and pathways (Table 2). Accordingly, the following sections will systematically elaborate on the effects of dandelion in terms of anti‐inflammation, antioxidant activity, and direct neuronal protection.

**TABLE 2:** Summary of in vivo/in vitro studies on the neuroprotective effects of *Taraxacum* extracts and their bioactive compounds.

Experimental materials (extracts/individual molecules)	Plant species (source)	Key molecular targets and signal pathways	Main research findings and conclusions	References
Neuroinflammation—Regulation of Cytokines				
Extract	Ethanol Extract of *T. officinale*	NF‐*κ*B, Nrf2/HO‐1 (Inhibiting COX‐2, iNOS)	Dose‐dependent inhibition of the release of TNF‐*α*, IL‐1*β* and IL‐6 in microglial cells; down‐regulation of the expression of inflammatory mediators.	Dong et al. (2020)
Extract	*T. coreanum*	Intercellular junction proteins (Tight Junctions)	Upregulate tight junction proteins to protect the integrity of the blood–brain barrier and prevent peripheral inflammation from invading the central nervous system.	Han et al. (2024)
Monomer (Chicoric acid)	Commercial reference standard	NF‐*κ*B	Inhibiting the NF‐*κ*B pathway can reduce memory impairment and amyloid protein production caused by systemic inflammation.	Liu, Chen, et al. (2017)
Monomer (Luteolin)	Commercial reference standard	M1/M2 polarization Nrf2‐ARE	Promote the polarization of microglia from the pro‐inflammatory M1 type to the anti‐inflammatory M2 type, thereby improving the inflammatory microenvironment.	Xu et al. (2014)
Neuroinflammation—Regulation of Key Inflammatory Signaling Pathways				
Extract	Ethanol Extract of *T. officinale*	MAPK, Nrf2/HO‐1	Actively activate the Nrf2 defense pathway and extensively inhibit the phosphorylation of MAPK family kinases, thereby multi‐levelly interfering with inflammatory signaling.	Huang et al. (2018)
Extract (Taraxasterol)	Commercial reference standard	LXR*α*, lipid raft (Lipid Rafts)	Stimulates the LXR*α* receptor, disassembles lipid rafts on the cell membrane, and interferes with the reception and transduction of upstream inflammatory signals.	Liu et al. (2018)
Monomer (Luteolin)	Commercial reference standard	NLRP3 inflammasome, Nrf2	Inhibit the assembly of the NLRP3 inflammasome, activate the Nrf2 pathway, and jointly exert anti‐inflammatory effects.	Zhang et al. (2021)
Neuroinflammation—Regulating the Activity of Glial Cells				
Extract	*T. officinale*	PI3K/Akt	Intervene in the PI3K/Akt pathway to exert overall anti‐inflammatory and neuroprotective effects	Nassan et al. (2018)
Extract	*T. coreanum* + Safflower Seeds	PI3K/Akt	Synergistically regulate the PI3K/Akt signaling pathway, reduce neuronal apoptosis induced by A*β*, and alleviate oxidative stress.	He et al. (2024)
Monomer (Chicoric acid)	Commercial reference standard	PDPK1/Akt/NF‐*κ*B NLRP3	Targeted promotion of the ubiquitination and degradation of the upstream kinase PDPK1, inhibition of Akt phosphorylation, and blocking of the downstream activation of NF‐*κ*B and NLRP3.	Zhang et al. (2023)
Oxidative stress—Metal chelators				
Extract	*T. officinale* roots and stems	Heavy metal adsorption (Cd, Zn)	Enrich and chelate heavy metals in the environment, immobilizing them in the surface tissues and reducing the entry of these metals into cells, thereby inhibiting the generation of reactive oxygen species.	Kano et al. (2021)
Monomer (Chlorogenic acid)	Commercial reference standard	Al^3+^ complexation	Strongly chelates aluminum ions, forming stable complexes that facilitate the excretion of aluminum from neurons and prevent cytotoxicity.	Wang et al. (2017)
Oxidative stress—Elimination of reactive oxygen species				
Extract	*T. officinale* leaf extract	Enzymatic defense system (SOD, CAT, GSH)	Restore the level of glutathione, enhance the activity of antioxidant enzymes, and systematically eliminate reactive oxygen species within cells.	Park et al. (2011)
Mixed metabolites	*T. kok‐saghyz* leaf metabolome extract	Flavonoid Biosynthesis	Enriched chamomolactone and luteolin derivatives directly scavenge ROS via their reducing activity, thereby alleviating neuronal oxidative stress.	Tan et al. (2025)
Monomer (Flavonoids)	Commercial reference standard	Hydrogen atom transfer (HAT) Phenolic hydroxyl group	Utilizing the reductive activity of phenolic hydroxyl groups, hydrogen atoms are provided, directly quenching reactive oxygen species and releasing stable phenoxide radicals.	Verma et al. (2012)
Oxidative stress—Inhibition of lipid peroxidation				
Extract	Extracts from the flowers, leaves and roots of *T. sect. Ruderalia*	Lipid oxidation chain reaction	Eliminate free radicals and reactive oxygen species, interrupt the lipid peroxidation chain reaction, reduce lipid oxidation damage to cell membranes, thereby exerting antioxidant protection effects.	Dias et al. (2014)
Extract	*T. officinale* flowers	Lipid oxidation chain reaction	Extend the lag period of lipid oxidation, reduce the rate of oxidative proliferation, and work synergistically with *α*‐tocopherol to inhibit the oxidation process.	Hu and Kitts (2005)
Monomer (Luteolin)	Commercial reference standard	Hydrogen atom transfer (HAT) Conjugated structure	The HAT mechanism provides hydrogen to terminate the lipid radical reaction; the conjugated structure stabilizes the radical and effectively inhibits peroxidation.	de Aguiar et al. (2025)
Oxidative stress—Enhancing endogenous defense				
Extract	*T. marginellum, T. hispanicum, T. lambinonii*	Endogenous antioxidant defense network	In a parallel comparative study, all three species consistently exhibited antioxidant capabilities in vitro and in vivo, effectively replenishing cellular antioxidant defenses and counteracting oxidative stress.	Muñoz Mingarro et al. (2015)
Extract (Taraxasterol)	Commercial reference standard	CYP2E1, Nrf2/HO‐1	Inhibiting CYP2E1 reduces ROS production, while activating the Nrf2/HO‐1 axis, and increasing GSH and SOD levels.	Xu et al. (2018)
Monomer (Chlorogenic acid isomers)	Commercial reference standard	Keap1‐Nrf2	Directly interfering with the Keap1‐Nrf2 interaction, promoting the nuclear translocation of Nrf2, and upregulating the expression of endogenous antioxidant enzymes (GCLC, SOD1).	Liang et al. (2019)
Protection of neurons‐Reduce A*β* deposition and Tau phosphorylation				
Extract	*T. coreanum* above‐ground part	BACE1, *γ*‐secretase (PS1, PS2)	Down‐regulate the expression of *β*‐secretase and *γ*‐secretase components, reduce the pathological processing of APP, and inhibit the generation of Aβ.	He et al. (2024)
Monomer (Luteolin)	Commercial reference standard	GSK‐3*α*, GSK‐3*β*	Selective inhibition of GSK‐3*α* interferes with A*β* production; inhibition of GSK‐3*β* reduces abnormal phosphorylation of Tau protein.	Sawmiller et al. (2014)
Protection of neurons‐Enhance mitochondrial function				
Extract	*T. mongolicum*	Mitochondrial membrane potential Glycolysis/TCA cycle	Inhibit the accumulation of ROS, stabilize the mitochondrial membrane potential, improve the energy metabolism process, and alleviate oxidative damage.	
Monomer (Chlorogenic acid)	Commercial reference standard	SIRT1/PGC‐1*α*/PPAR*γ* BACE1	Activating the SIRT1/PGC‐1*α* axis promotes mitochondrial biogenesis; inhibiting BACE1 reduces the toxicity of A*β* to mitochondria.	Shi et al. (2023)

### 3.1 Neuroinflammation

Inflammation of the central nervous system is referred to as neuroinflammation (Kent et al. 2020) and is primarily mediated by glial cells (Leng and Edison 2021), particularly microglia and astrocytes. As the resident innate immune cells of the brain, microglia continuously monitor the neural environment and maintain homeostasis (Gu et al. 2021). They can be activated by stimuli such as amyloid‐*β* (*Aβ*), lipopolysaccharide (LPS), and interferons, subsequently polarizing into the pro‐inflammatory M1 phenotype or the anti‐inflammatory M2 phenotype. M1 microglia release pro‐inflammatory cytokines—including interleukin‐1*β* (IL‐1*β*), tumor necrosis factor‐*α* (TNF‐*α*), and interleukin‐6 (IL‐6)—as well as nitric oxide (NO), thereby exacerbating inflammation and impairing neuronal function. In contrast, M2 microglia enhance phagocytosis and promote neuronal protection through the secretion of anti‐inflammatory cytokines such as interleukin‐4 (IL‐4), interleukin‐10 (IL‐10), and arginase‐1 (Tang and Le 2016). Astrocytes also contribute to neuroinflammation by releasing factors such as IL‐6, TNF‐*α*, IL‐1*β*, and complement component C3, which can damage neurons. However, peripheral immune cells, including T cells, lymphocytes, and monocytes, may also exert neuroprotective effects (Figure 2).

Under pathological conditions, neuroinflammation becomes chronic, characterized by sustained release of pro‐inflammatory mediators and reduced production of anti‐inflammatory factors. This imbalance exacerbates synaptic dysfunction, neuronal death, and impaired neurogenesis (Wang et al. 2019). Therefore, modulation of cytokine levels, inflammatory signaling pathways, and glial cell activation states represents a key strategy for suppressing neuroinflammation.

#### 3.1.1 Regulation of Cytokines

Exposure to external stimuli activates signaling pathways that regulate the production of pro‐ and anti‐inflammatory cytokines. Pro‐inflammatory cytokines, such as TNF‐*α*, IL‐1*β*, IL‐6, and IL‐8, promote the activation of neutrophils and lymphocytes, increase vascular endothelial permeability, modulate tissue metabolism, and stimulate the production of additional inflammatory mediators. In contrast, anti‐inflammatory cytokines–including IL‐4, IL‐5, and IL‐10–facilitate lymphocyte proliferation, enhance antibody production, and suppress macrophage activity. Together, these cytokines maintain immune homeostasis.

Numerous studies demonstrate that dandelion extracts exhibit significant immunomodulatory effects in neuroinflammatory models. For example, ethanol extracts of *Taraxacum officinale* have been shown to dose‐dependently inhibit the release of pro‐inflammatory cytokines such as TNF‐*α*, IL‐1*β*, and IL‐6 in microglial cells, while downregulating the expression of inducible nitric oxide synthase (iNOS) and cyclooxygenase‐2 (COX‐2). These effects are mediated through the inhibition of the NF‐*κ*B signaling pathway and activation of the Nrf2/HO‐1 pathway, indicating a synergistic multi‐target mechanism (Dong et al. 2020). Similarly, *Taraxacum coreanum* extracts have been reported to preserve blood–brain barrier integrity by upregulating tight junction proteins, thereby limiting the infiltration of peripheral inflammatory factors into the central nervous system (Han et al. 2024). At the molecular level, studies on isolated compounds provide further mechanistic insights. For instance, chicoric acid reduces the production of inflammatory mediators by inhibiting NF‐*κ*B signaling (Liu, Chen, et al. 2017), while luteolin promotes the polarization of microglia from the pro‐inflammatory M1 phenotype to the anti‐inflammatory M2 phenotype, thereby facilitating the restoration of the inflammatory microenvironment (Xu et al. 2014). Overall, studies on dandelion extracts provide direct evidence of their anti‐inflammatory efficacy, whereas investigations of individual bioactive compounds elucidate the underlying molecular mechanisms. Together, these complementary findings highlight the neuroprotective potential of dandelion.

#### 3.1.2 Regulation of Key Inflammatory Signaling Pathways

NF‐*κ*B is a family of transcription factors that typically exist as an active heterodimer composed of P50 and P65 subunits and is ubiquitously expressed in eukaryotic cells (Bao et al. 2020). In the resting state, NF‐*κ*B is bound to the inhibitory protein I*κ*B and remains inactive in the cytoplasm. Upon stimulation by extracellular signals, I*κ*B kinase (IKK) is activated, leading to phosphorylation and degradation of I*κ*B. This releases NF‐*κ*B, enabling its translocation into the nucleus, where it induces the transcription of pro‐inflammatory genes, including TNF‐*α*, IL‐1*β*, and IL‐6. Modulation of this pathway—through inhibition of IKK activity, stabilization of I*κ*B, or prevention of NF‐*κ*B binding—can suppress NF‐κB nuclear translocation and downstream gene expression. Consequently, this reduces the production of pro‐inflammatory cytokines, enhances the anti‐inflammatory responses, and restores immune balance (Yi et al. 2019).

Studies on dandelion extracts demonstrate their multi‐target regulatory effects on key inflammatory pathways. For instance, ethanol extracts of *Taraxacum officinale* activate the Nrf2/HO‐1 antioxidant defense pathway in neuronal models while simultaneously inhibiting phosphorylation of MAPK family kinases in microglial models, thereby modulating inflammatory signaling at multiple levels (Huang et al. 2018). At the molecular level, investigations of specific bioactive compounds provide deeper mechanistic insights. Taraxasterol has been shown to interfere with upstream signal transduction by activating the LXR*α* receptor and disrupting cell membrane lipid rafts (Liu et al. 2018). Similarly, luteolin exerts anti‐inflammatory effects by inhibiting NLRP3 inflammasome assembly while activating the Nrf2 pathway (Zhang et al. 2021). Overall, dandelion extracts exhibit broad, multi‐pathway regulatory activity, whereas studies on individual compounds elucidate the precise molecular mechanisms underlying these effects.

#### 3.1.3 Regulating Glial Cell Activations

A hallmark of neuroinflammation in neurodegenerative diseases is the activation of glial cells, particularly microglia and astrocytes, which release pro‐inflammatory cytokines, chemokines, and other neurotoxic factors (Singh et al. 2025). Modulating glial cell activity–especially promoting the transition of microglia from the pro‐inflammatory M1 phenotype to the anti‐inflammatory M2 phenotype–is a key therapeutic strategy.

Evidence suggests that dandelion‐derived compounds exert anti‐inflammatory effects by targeting signaling pathways such as PI3K/Akt (Nassan et al. 2018). Direct evidence from extract‐based studies shows that, in an Alzheimer's disease model, a combined extract of *Taraxacum coreanum* and safflower seed produces synergistic effects, reducing A*β*‐induced neuronal apoptosis and oxidative stress via modulation of the PI3K/Akt pathway (He et al. 2024). At the mechanistic level, studies on isolated compounds provide further precision. Chicoric acid, a major phenolic constituent, has been shown to promote ubiquitination and degradation of the upstream kinase PDPK1, thereby inhibiting Akt phosphorylation (Thr308) and suppressing downstream NF‐κB activation and NLRP3 inflammasome assembly. This ultimately mitigates mitochondrial dysfunction and oxidative stress (Zhang et al. 2023). In summary, dandelion extracts demonstrate overall efficacy in regulating glial cell activation, while studies on individual compounds clarify the underlying molecular targets and pathways.

Neuroinflammation is driven primarily by glial cell activation within the central nervous system. Under pathological conditions, sustained activation leads to excessive release of pro‐inflammatory mediators, reduced anti‐inflammatory responses, synaptic dysfunction, neuronal death, and impaired neurogenesis. Therefore, targeting cytokine regulation, inflammatory signaling pathways, and glial cell activation reflects an effective strategy for alleviating neuroinflammation.

### 3.2 Oxidative Stress

OS, a major driver of cellular apoptosis, tissue damage, and pathological changes in vivo (Lv et al. 2022), disrupts intracellular signaling pathways and is closely associated with inflammation (Wang et al. 2022). It is a complex process often accompanied by mitochondrial dysfunction and impaired mitophagy, which further increase the production of reactive oxygen species (ROS) and reactive nitrogen species (RNS), thereby exacerbating oxidative damage (Gkekas et al. 2021). OS‐related diseases arise through several key mechanisms. First, excessive ROS and RNS react with intracellular lipids, proteins, and nucleic acids, leading to lipid peroxidation, protein oxidation, and structural and functional cellular damage. Peroxynitrite, formed by the reaction of nitric oxide (NO) with mitochondrial superoxide, is a major contributor to oxidative injury (Dash et al. 2024). Second, disruption of the antioxidant defense system—including reduced activity of enzymatic and non‐enzymatic antioxidants—impairs the clearance of ROS. Additionally, dysregulated redox signaling alters multiple biological processes by modifying proteins, promoting inflammation, inducing apoptosis, regulating autophagy, and impairing mitochondrial function (Figure 2) (Sies and Jones 2020).

Under pathological conditions, this imbalance results in sustained ROS overproduction alongside diminished endogenous antioxidant capacity. Excessive ROS not only damages biomolecules, but also activates apoptotic signaling pathways, accelerating cellular aging and death. Furthermore, ROS accumulation can trigger abnormal activation of transition metal ions (e.g., iron and copper), generating highly reactive hydroxyl radicals that further amplify oxidative damage. Therefore, strategies such as metal chelation, ROS scavenging, inhibition of lipid peroxidation, and enhancement of endogenous antioxidant defenses are critical for mitigating OS and interrupting this cycle of damage.

#### 3.2.1 Metal Chelation

Metal ions such as Cu, Fe, Mn, and Zn are essential for maintaining physiological functions and homeostasis. Among these, iron is the most abundant metal in the brain, followed by copper and zinc (Liu et al. 2019). These metals participate in various signaling processes; for instance, zinc deficiency can influence A*β* aggregation (Mezzaroba et al. 2019). However, dysregulated metal homeostasis contributes to oxidative stress, making metal chelation a widely recognized antioxidant strategy (Gulcin and Alwasel 2022). Flavonoids, for example, can chelate transition metals such as Fe^2+^ and Cu^2+^, typically in a 2:1 stoichiometric ratio, through hydroxyl and carbonyl groups (Kejík et al. 2021). Their chelation capacity is influenced by factors such as pH, solvent properties, stoichiometry, and redox potential, although the number and position of hydroxyl groups remain the primary determinants (Kejík et al. 2021).

Dandelion exhibits notable metal‐chelating capacity, contributing to its antioxidant effects. Evidence indicates that dandelion can accumulate and sequester heavy metals such as cadmium and zinc, often immobilizing them in surface tissues (e.g., roots and stems), thereby limiting cellular uptake and reducing ROS generation (Kano et al. 2021). At the molecular level, studies on individual compounds provide further insight. Chlorogenic acid, a key phenolic component of dandelion, has been identified as an effective chelator of aluminum ions. It forms stable complexes that facilitate aluminum excretion from neurons, thereby reducing aluminum‐induced cytotoxicity (Wang et al. 2017). In summary, studies on whole dandelion plants demonstrate their capacity for heavy metal sequestration, while investigations of individual compounds elucidate the underlying molecular mechanisms of chelation and detoxification.

#### 3.2.2 Elimination of ROS

ROS are chemically reactive molecules generated during the incomplete reduction of oxygen and act as key mediators of inflammation (D'Autreaux and Toledano 2007). Major ROS include hydroxyl radicals (OH), superoxide anions (O_2_^−^), singlet oxygen, and hydrogen peroxide (H_2_O_2_) (Dickinson and Chang 2011). Excessive ROS can attack biomolecules such as lipids, proteins, and DNA, leading to oxidative damage that accumulates over time, accelerating aging and contributing to the development of diseases including cancer, cardiovascular disorders, neurodegenerative diseases, respiratory conditions, and digestive disorders. When ROS levels exceed the capacity of the antioxidant defense system, OS is triggered, promoting cell death and driving pathological processes (Yang and Lian 2020). Flavonoids play an important role in ROS scavenging, largely due to structural features such as hydroxyl groups on the B ring and conjugation between the C2 = C3 double bond and the C4 carbonyl group. These features enable flavonoids to donate hydrogen atoms or electrons to neutralize ROS, forming relatively stable radicals; the stability of these radicals determines subsequent reaction pathways (Agati et al. 2020).

Studies indicate that dandelion extracts effectively enhance cellular antioxidant defense systems. For example, methanol and aqueous extracts of dandelion leaves can restore lipopolysaccharide (LPS)‐induced depletion of glutathione (GSH) and increase the activity of key antioxidant enzymes such as superoxide dismutase and catalase, thereby facilitating the clearance of intracellular ROS. The methanol extract exhibits stronger activity, likely due to its higher content of total phenolics, luteolin, and chicoric acid (Park et al. 2011). Recent metabolomic analyses further support the presence of a broad antioxidant network in dandelion. Leaves of *Taraxacum kok‐saghyz* are enriched in chicoric acid and luteolin derivatives through flavonoid biosynthetic pathways, reinforcing their ROS‐scavenging capacity and ability to enhance endogenous defense systems (Tan et al. 2025). At the molecular level, studies on individual compounds provide additional insight. Flavonoids, through the redox activity of their phenolic hydroxyl groups, can donate hydrogen atoms and form phenoxyl radicals, thereby protecting cells from ROS‐induced damage (Verma et al. 2012). Overall, dandelion extracts demonstrate synergistic ROS‐scavenging effects via enhancement of enzymatic antioxidant systems, while studies on individual flavonoids elucidate the underlying mechanisms of electron and hydrogen atom transfer.

#### 3.2.3 Inhibition of Lipid Peroxidation

Lipid peroxidation is a chain reaction involving the formation and propagation of lipid free radicals, oxygen incorporation, rearrangement of unsaturated lipid bonds, and eventual degradation of membrane lipids. It is widely regarded as a major molecular mechanism underlying oxidative cellular damage and cell death (Chakraborty et al. 2024). This process is typically initiated by environmental and chemical factors, such as temperature and the composition of unsaturated fatty acids, and proceeds through three stages: initiation, propagation, and termination. Reactive species, particularly •OH, abstract hydrogen atoms from methylene‐interrupted carbons in polyunsaturated fatty acids (PUFAs), generating alkyl radicals. The susceptibility of fatty acids to oxidation increases with the number of double bonds. During the initiation phase, enzymes and metal ions promote ROS formation, accelerating the reaction, while alkyl radicals may undergo isomerization to form conjugated double bonds (Gutiérrez‐del‐Río et al. 2021).

Dandelion has been shown to inhibit lipid peroxidation by interrupting this oxidative chain reaction. For example, wild *Taraxacum* sect. *Ruderalia* is rich in phenolic compounds that scavenge free radicals and ROS, thereby blocking the propagation phase and reducing membrane oxidative damage (Dias et al. 2014). Experimental evidence further demonstrates that dandelion flower extracts can prolong the lag phase of lipid oxidation, reduce the rate of propagation, and scavenge reactive species such as superoxide anions. Key constituents, including luteolin and caffeic acid, may also act synergistically with α‐tocopherol to enhance inhibition of lipid peroxidation (Hu and Kitts 2005). At the molecular level, studies on individual compounds provide mechanistic insight. Luteolin, for instance, can donate hydrogen atoms from its phenolic hydroxyl groups to lipid radicals via a hydrogen atom transfer mechanism, thereby terminating the chain reaction. Its conjugated structure stabilizes the resulting phenoxyl radicals, enhancing its antioxidant efficiency (de Aguiar et al. 2025). Overall, dandelion extracts demonstrate effective inhibition of lipid peroxidation at the system level, while studies on specific compounds such as luteolin elucidate the underlying molecular mechanisms involving electron and hydrogen atom transfer.

#### 3.2.4 Enhancing the Endogenous Antioxidant Defense System

Lipid peroxidation, involving the generation and propagation of lipid free radicals, oxygen uptake, and rearrangement of unsaturated lipid bond patterns, ultimately leads to the degradation of membrane lipids. This process is widely recognized as a major molecular mechanism underlying cellular oxidative damage and cell death (Chakraborty et al. 2024). The body produces compounds that induce the formation of endogenous antioxidants (such as GSH) and antioxidant enzymes in response to OS (Young et al. 2010). These endogenous antioxidants act synergistically to eliminate excess free radicals, mitigate oxidative damage, restore redox homeostasis, and protect against OS.

In a comparative study, the three species *T. marginellum*, *T. hispanicum*, and *T. lambinonii* consistently exhibited both in vitro and in vivo antioxidant activities, effectively replenishing and enhancing basal cellular antioxidant defenses and counteracting oxidative stress–induced deterioration (Muñoz Mingarro et al. 2015). Further studies indicate that specific components of dandelion can activate cellular self‐protective mechanisms. At the molecular level, studies on standardized compounds reveal key pathways of action. For example, taraxasterol can inhibit CYP2E1 to reduce ROS generation while activating the Nrf2/HO‐1 signaling pathway, thereby increasing GSH levels and superoxide dismutase (SOD) and strengthening antioxidant defenses (Xu et al. 2018). Similarly, chlorogenic acid and its isomers (e.g., dicaffeoylquinic acid) have been shown to interfere with the Keap1‐Nrf2 interaction, promote Nrf2 nuclear translocation, and upregulate the expression of endogenous antioxidant enzymes (e.g., GCLC, SOD1). Notably, structurally more complex isomers exhibit stronger activation effects (Liang et al. 2019). These findings indicate that sterol and phenolic acid components in dandelion enhance intrinsic antioxidant capacity through regulation of the Nrf2 pathway.

OS is a major driver of cell apoptosis, tissue damage, and pathological changes. It disrupts intracellular signaling pathways and contributes to inflammation. Its pathological state arises from sustained ROS overproduction coupled with reduced endogenous antioxidant defenses. Excessive ROS can trigger lipid peroxidation, activate apoptotic pathways, and cause widespread cellular damage. However, OS can be mitigated through metal chelation, ROS scavenging, inhibition of lipid peroxidation, and enhancement of endogenous antioxidant systems, thereby disrupting the cycle of cellular damage.

### 3.3 Protection of Neurons

An axon is an elongated projection of a neuronal cell that conducts electrical impulses from one nerve cell to another, facilitating cellular communication. Axonal damage or injury is common in various neurodegenerative diseases and often results in neuronal damage and cell death (Patel et al. 2020). Neuronal regeneration effectively restores brain cognitive consciousness and nervous system function and is the most effective strategy for improving and treating AD. Therefore, brain function can be restored by protecting neurons.

#### 3.3.1 Reducing Aβ Deposits and Excessive Tau Protein Phosphorylation

A*β* is generated by neurons and astrocytes in the brain, accumulates in the extracellular space, and is subsequently cleared via the cerebrospinal fluid‐lymphatic pathway and the vascular network (Brothers et al. 2018). In the extracellular aqueous environment, A*β* adopts a random coil structure, whereas in membrane‐associated environments, it assumes a helical conformation with kinks (Mandal and Pettegrew 2004). Tau, a microtubule‐binding protein, is essential for maintaining neuronal structure and integrity (Roy et al. 2023).

In reducing A*β* deposition and abnormal tau phosphorylation, studies have demonstrated the multi‐target neuroprotective potential of dandelion and its components. Direct evidence shows that aerial part extracts of *Taraxacum coreanum* can downregulate the expression of *β*‐secretase (BACE) and *γ*‐secretase components (PS1, PS2), thereby reducing the pathological processing of amyloid precursor protein (APP) and inhibiting A*β* generation. In addition, these extracts modulate apoptosis‐related proteins, alleviating A*β*‐induced neurotoxicity (He et al. 2024). At the mechanistic level, studies on individual compounds provide more precise insights. For example, luteolin has been shown to selectively inhibit GSK‐3*α* activity, disrupting the interaction between presenilin 1 and APP to reduce A*β* production. It can also inhibit kinases such as GSK‐3*β*, reduce abnormal tau phosphorylation, and attenuate neuroinflammation, thereby alleviating Alzheimer's disease‐like pathology through multiple pathways (Sawmiller et al. 2014). Overall, studies on dandelion extracts demonstrate their ability to regulate A*β* generation and tau phosphorylation, while investigations of compounds such as luteolin clarify the underlying molecular mechanisms at the level of kinases and protein interactions.

#### 3.3.2 Enhancing Mitochondrial Function

Mitochondria, often referred to as the “powerhouses” of the cell, play a critical role in neuronal function and survival as the primary energy‐producing organelles. Impairment of mitochondrial function can lead to neuronal damage and death, contributing to the development of neurological disorders. Mitochondria are also involved in the regulation of neuronal death and in cellular defense against various stresses (Nicholls and Budd 2000). Flavonoids can enhance mitochondrial function, facilitate the clearance of A*β* deposits, and improve synaptic plasticity, thereby contributing to neuroprotection in Alzheimer's disease.

Studies indicate that dandelion extracts help maintain neuronal energy metabolism and protect against oxidative damage. For example, dandelion extract has been shown to inhibit H_2_O_2_‐induced ROS accumulation, stabilize mitochondrial membrane potential, and improve metabolic processes such as glycolysis and the tricarboxylic acid cycle, thereby reducing oxidative stress‐induced neuronal apoptosis (Hu and Kitts 2005). At the mechanistic level, studies on individual compounds reveal specific regulatory pathways. Chlorogenic acid, for instance, enhances mitochondrial biogenesis and function by activating the SIRT1/PGC‐1*α*/PPAR*γ* signaling pathway, while also inhibiting BACE1 activity to reduce A*β‐*induced mitochondrial toxicity. Additionally, it promotes mitochondrial homeostasis by increasing the activity of antioxidant enzymes such as superoxide dismutase (SOD) (Shi et al. 2023). In summary, dandelion extracts exhibit overall protective effects on mitochondrial function and energy metabolism, while studies on compounds such as chlorogenic acid provide insight into the underlying molecular mechanisms.

**FIGURE 3:**
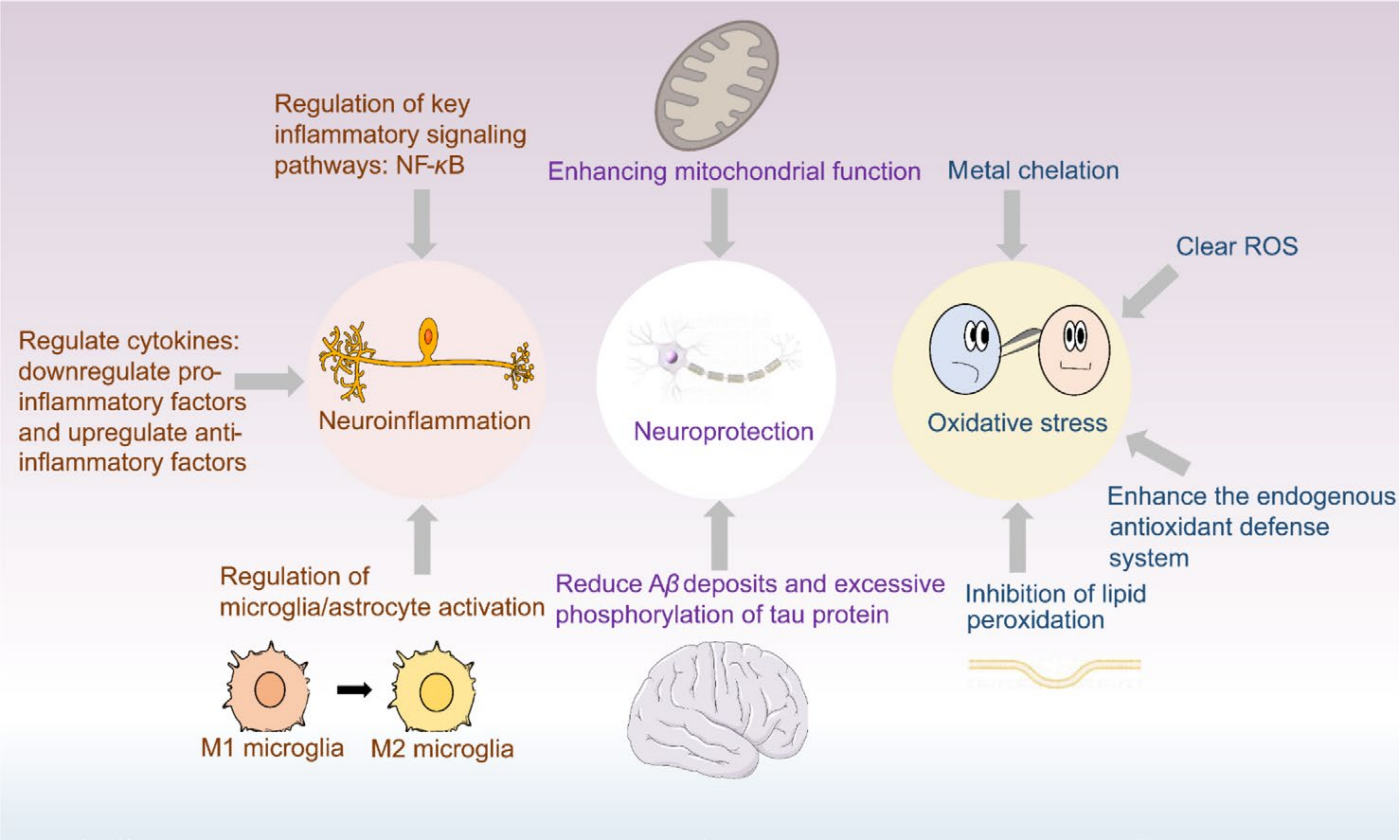
The active ingredients in dandelion intervene in neuroinflammation and oxidative stress through multidimensional mechanisms, exerting a neuroprotective effect.

In summary, flavonoids, phenolic acids, sterols, and terpenoids play multiple roles in neuroprotection. In neuroinflammation, they can modulate pro‐inflammatory and anti‐inflammatory cytokines, key inflammatory pathways, and glial cell activation. In response to OS, they can chelate metals, scavenge ROS, inhibit lipid peroxidation, and enhance endogenous antioxidant defenses. In protecting neurons, they can reduce A*β* deposition and tau hyperphosphorylation and also enhance mitochondrial function (Figure 3).

## 4 Structure–Activity Relationship of Flavonoids, Phenolic Acids, Terpenoids and Sterols in Dandelion

**FIGURE 4:**
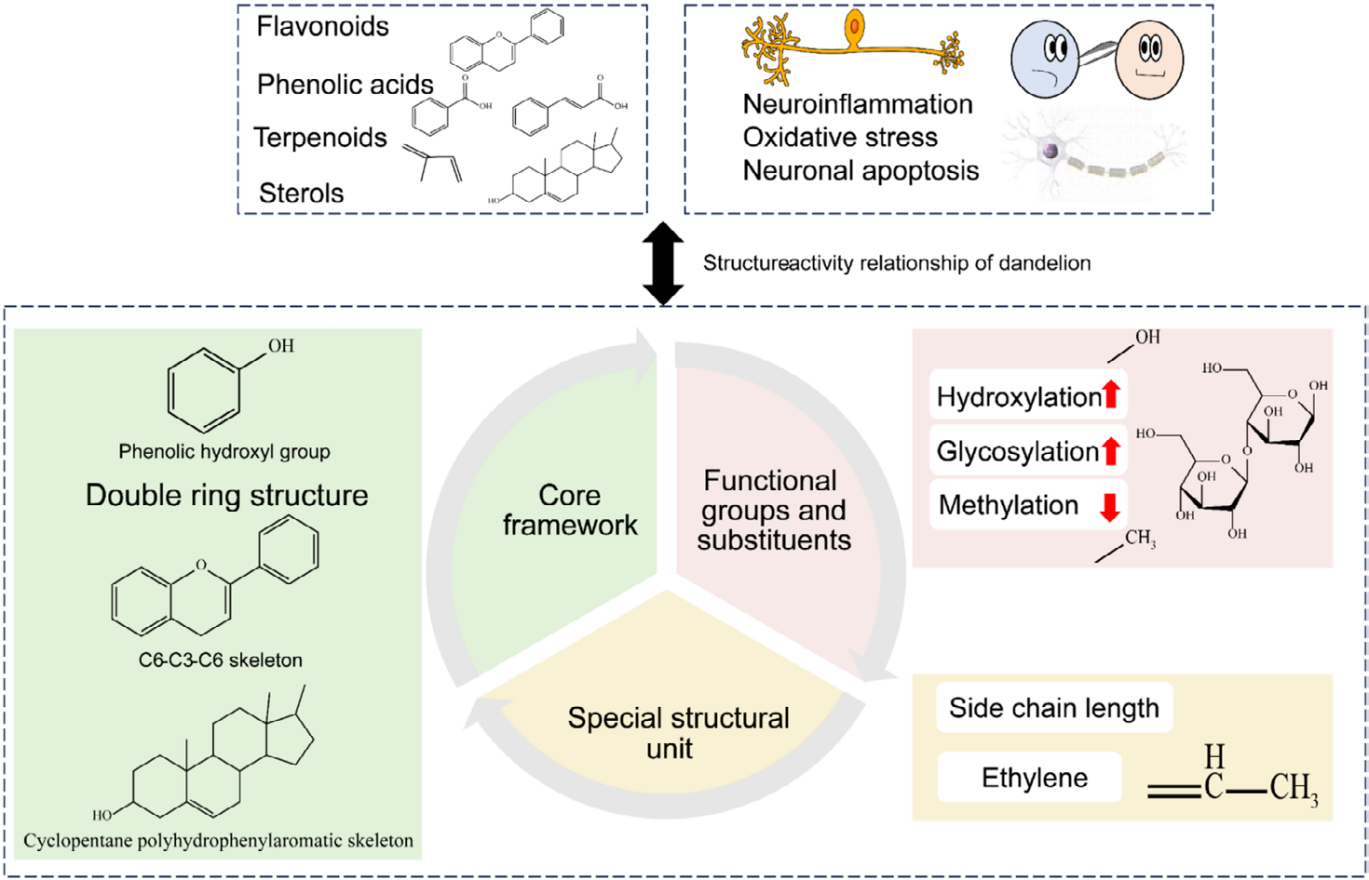
Structure–activity relationship of active components in dandelion.

As a plant that is both medicinal and edible, dandelion has attracted significant attention due to the notable antioxidant, anti‐inflammatory, and neuroprotective activities of the flavonoids, phenolic acids, sterols, and terpenoids it contains. Figure 4 illustrates the structure–activity relationships between the structural characteristics of these components and their biological effects, providing a theoretical basis for elucidating the material basis of their pharmacological actions and developing their functional constituents.

### 4.1 Structure–Activity Relationships of Core Backbones

The core skeleton serves as the fundamental structure of an active compound, defining the category and baseline biological activity. Different skeletons influence target recognition and binding related to neuroinflammation and OS through their distinct spatial conformations.

In phenolic acids, the aromatic ring backbone often features ortho‐positioned phenolic hydroxyl groups that form intramolecular hydrogen bonds, stabilize free radical intermediates, and significantly enhance antioxidant capacity compared to meta‐substituted structures (Chen et al. 2020). Among terpenoids, the rigid bicyclic oplopane backbone of *Tussilago farfara* sesquiterpenes exhibits stronger anti‐inflammatory and antioxidant effects than the more flexible chain‐like bisabolane backbone (Lim et al. 2015). The C6–C3–C6 backbone of flavonoids influences molecular planarity and electron delocalization through double bonds and keto groups, serving as a key structural basis for antioxidant and anti‐inflammatory activities (Wang et al. 2018b). The steroidal backbone of sterols (cyclopentanoperhydrophenanthrene) is highly hydrophobic, facilitating membrane penetration. It can target mitochondria to inhibit ROS production and activate the Nrf2 pathway, thereby reducing OS (He et al. 2020). Additionally, it can interact with membrane‐associated receptors or intracellular signaling proteins such as NF‐*κ*B and MAPK to suppress neuroinflammation (Chen et al. 2019).

The principle that a compound's core scaffold determines its bioactivity is well illustrated by dandelion triterpenoids. A representative example is taraxasterol, whose activity arises from a subtle but critical skeletal modification. While both taraxasterol and the common *α*‐amyrin are derived from the ursane‐type pentacyclic skeleton, they differ at a key position. *α*‐Amyrin contains a conventional endocyclic double bond between C‐12 and C‐13, whereas taraxasterol—a chemotaxonomic marker of *Taraxacum*—features a distinctive exocyclic methylene group (=CH_2_) at C‐20 (30) on the E‐ring (Jiao et al. 2022). This structural difference, although minor, has significant functional consequences. Computational docking and pharmacological studies indicate that the exocyclic double bond creates a distinct steric and electronic profile, enhancing taraxasterol's affinity for key inflammatory signaling targets. Specifically, it promotes stronger binding to the ATP‐binding pocket of I*κ*B kinase *β* and to the Toll‐like TLR4 complex compared to its analogous structures lacking this feature. As a result, taraxasterol exhibits enhanced inhibition of LPS‐induced NF‐*κ*B pathway activation (Zhang et al. 2014). These findings indicate that the C‐20 exocyclic methylene group functions as a critical pharmacophore contributing to the enhanced anti‐inflammatory activity of dandelion‐derived triterpenes.

### 4.2 Structure–Activity Relationships of Functional Groups and Substituents

Functional groups and substituents regulate biological activity through electronic effects, steric hindrance, and polarity. The number of hydroxyl groups influences electron transfer efficiency and determines antioxidant capacity. Modifications such as glycosylation alter target binding due to steric hindrance, while polarity regulates molecular distribution in neural tissue. Together, these factors form a finely tuned regulatory system governing biological activity.

Antioxidant activity correlates with the total number of hydroxyl substituents (Woodman et al. 2005). Hydroxyl groups directly scavenge ROS via hydrogen atom transfer and can also enhance the synthesis of endogenous antioxidants. Additionally, they form hydrogen bonds with the polar heads of phospholipids, stabilizing membrane structures and indirectly inhibiting signaling pathways such as NF‐*κ*B (Liu et al. 2016). Structural features such as the 3′,4′‐dihydroxy groups in the B ring, the 2,3‐double bond conjugated with the 4‐keto group in the C‐ring, and the 3‐hydroxy group contribute to the antioxidant capacity of flavonoids (Zhang et al. 2022). Caffeic acid, which contains two phenolic hydroxyl groups, exhibits stronger antioxidant activity and A*β* inhibition than ferulic acid, which has only one phenolic hydroxyl group (Chen et al. 2017).

Polar functional groups, including hydroxyl and carboxyl groups, enhance binding affinity to inflammatory targets through hydrogen bonding and electrostatic interactions. For example, chicoric acid, containing multiple phenolic hydroxyl and carboxyl groups along with a conjugated double bond, binds more effectively to TLR4 and inhibits TLR4‐MD‐2 complex formation compared to chlorogenic or caffeic acid, thereby enhancing its anti‐inflammatory activity (Zou et al. 2023). Ortho‐dihydroxyphenyl groups further improve radical‐scavenging efficiency; for instance, quercetin, with an ortho‐catechol structure, exhibits stronger ROS‐scavenging ability than its hydroxyl‐deficient metabolites (Pavlica and Gebhardt 2010).

Glycosylation reduces molecular lipophilicity, limiting direct interaction with free radicals and affecting the stability of conjugated systems, thereby decreasing antioxidant activity. It may also alter molecular conformation, reducing binding affinity to inflammation‐related targets and weakening anti‐inflammatory effects (Wang et al. 2018a). Similarly, methoxy substitution on flavonoid rings reduces antioxidant activity by disrupting molecular planarity.

The introduction of a methyl group at the 5‐position of the furan ring in the monoterpene isopulegone enhances interactions with polar groups, resulting in increased inhibition of NO and MCP‐1, as well as enhanced suppression of NF‐*κ*B and AP‐1 pathways (Souza et al. 2014).

Sesquiterpene lactones (SLs), responsible for the characteristic bitterness of dandelion, constitute a major class of its bioactive compounds. Their activity depends on the presence of an *α*, *β*‐unsaturated‐*γ*‐lactone ring, which acts as a Michael acceptor and enables covalent modification of cellular nucleophiles such as cysteine residues in target proteins. However, the nature and position of additional functional groups on the germacranolide skeleton critically influence biological outcomes. A comparison between taraxinic acid (TA) and parthenolide (PTL) illustrates this principle. PTL contains a highly reactive C1–C10 epoxide group in addition to the *α*‐methylene‐*γ*‐lactone, conferring strong cytotoxic activity and inducing apoptosis in cancer cells (e.g., HL‐60 leukemia cells, IC_50_ ≈ 1–5 μM) primarily through oxidative stress and mitochondrial dysfunction (Guzman et al. 2005). In contrast, TA lacks this epoxide group while retaining other structural features (Jung‐Hye Choi et al. 2002). This difference significantly alters its activity: rather than inducing apoptosis, TA promotes differentiation of HL‐60 cells into monocyte/macrophage‐like phenotypes by downregulating c‐myc and upregulating p21CIP1 and p27KIP1 (Renna 2026). Thus, the presence or absence of the epoxide group functions as a molecular switch, shifting the activity from cytotoxicity to regulation of cell differentiation, and suggesting a potentially broader therapeutic window for dandelion‐derived SLs such as TA.

### 4.3 Structure–Activity Relationships of Special Structural Units

Special structural units play a key regulatory role in neuroprotection due to their unique spatial adaptability and electronic interaction modes.

The length of the alkyl side chain in ferulic acid correlates with its ability to inhibit acetylcholinesterase and butyrylcholinesterase. A five‐carbon chain optimally matches the distance between the catalytic active site and the peripheral anionic site of cholinesterases (ChEs), enabling dual‐site binding and maximal activity. Longer or shorter chains reduce activity due to spatial mismatch (Lan et al. 2020). The ethylidene group on the R3 side chain of sterol compounds increases the electron density of antioxidant groups through conjugation, thereby enhancing antioxidant properties as its content increases.

Beyond modifications of monomeric units, some dandelion compounds derive enhanced activity from unique conjugated structural motifs. Chicoric acid is a representative dandelion metabolite formed by esterification of two caffeic acid molecules with a central tartaric acid moiety. While caffeic acid itself is an effective antioxidant, chicoric acid exhibits distinct and more specific pharmacological activities, including inhibition of the HIV‐1 integrase and the Yersinia virulence factor tyrosine phosphatase (YopH) (King et al. 1999). This enhanced activity is attributed to its dimeric structure. Molecular modeling studies indicate that the tartaric acid linker maintains the two catechol (o‐dihydroxyphenyl) groups at a defined distance and orientation (Kuban‐Jankowska et al. 2016). This arrangement creates a bidentate ligand geometry, enabling simultaneous interaction with the catalytic active site and an adjacent binding pocket of target enzymes—an interaction not achievable by monomeric caffeic acid. Thus, tartaric acid‐mediated dimerization serves as a key structural determinant that enhances specificity and biological activity, illustrating how unique structural units can confer novel functional properties.

In summary, studies on the structure–activity relationships of flavonoids, phenolic acids, terpenoids, and sterols in dandelions have demonstrated that the core skeleton is the primary factor determining their biological activity. Functional groups and substituents can exert regulatory effects through electronic and steric interactions, whereas unique structural units are important because of their spatial or electronic benefits. These research findings provide highly valuable guidance for designing highly active bioactive molecules.

## 5 Applications of Dandelion

**FIGURE 5:**
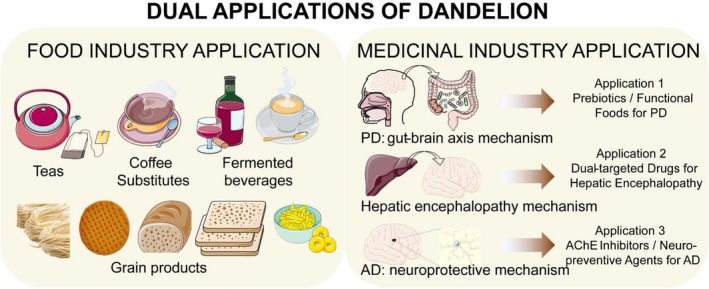
Applications of dandelion in food and medicine.

Dandelion is a common wild plant widely used in food and medicine. Its application history can be traced back to ancient times, and it has various uses in different cultures. Although dandelion is widely recognized for its medicinal properties, it has also found successful use in the global food industry as a safe and edible plant. The United States Food and Drug Administration has listed it as a safe food for people with rare allergies (Martinez et al. 2015). Figure 5 summarizes the applications of dandelions in food and medicine.

### 5.1 Application of Dandelion in the Food Industry

In the Qing Dynasty, Chinese herbalists acknowledged the dual use of dandelion. Wang Mengying, in “Suixiju Diet Spectrum,” noted, “Young plants serve as vegetables, while mature plants serve as medicine” while referring to dandelion. This tradition persists, with dandelion stems and leaves consumed raw, cold, or fried, making them herbs of both culinary and medicinal origin. Additionally, dandelion's versatility is recognized in various food products (Wu et al. 2025).

#### 5.1.1 Beverages

Dandelion leaves possess not only culinary appeal but also substantial nutritional value, enabling their transformation into functional beverages such as herbal teas and coffee analogues (Wirngo et al. 2016). Notably, fermentation processes have been proven to enhance the bioavailability of bioactive compounds. For instance, (Wu et al. 2016) optimized the production of dandelion root compound wine through orthogonal experiments, identifying key parameters. The nine‐step protocol, including enzymatic hydrolysis and yeast fermentation, yielded a light‐yellow wine with reduced bitterness and retained bioactivity (14.7% alcohol, 6.85 g/L acidity). Further, (Kim et al. 2015) developed a probiotic drink using *Lactobacillus acidophilus* F‐46 to ferment dandelion extract, and the fermentation process increased the caffeic acid concentration of the dandelion drink by 4.3 times by increasing the cinnamesterase activity. This highlights the role of bacterial enzyme conversion in the release of bound phenols, thereby enhancing antioxidant capacity. This fermented beverage proved that Lactobacillus fermentation can effectively enhance the bioactive function of dandelion beverage. Additionally, the dried leaves of dandelion can be used as a common ingredient in various digestive aids, dietary drinks, and herbal beers, and are widely used in the United Kingdom and Canada (González‐Castejón et al. 2012), Its roasted roots are used as a substitute for coffee (Dias et al. 2014).

#### 5.1.2 Grain Products

As a nutrient‐dense botanical, dandelion is increasingly utilized in grain‐based functional foods. (Cacak‐Pietrzak et al. 2023) found that the addition of dried dandelion flowers (DF) to wheat flour enhanced the mineral, fiber, fat content and antioxidant capacity of the bread but decreased the volume and lightness of the bread, increased the hardness and yellowness, and affected the sensory acceptability. Therefore, it is recommended that DF be added in an amount not exceeding 2%–3% of wheat flour to achieve the optimum balance. Similarly, Ra and Kim (2017) developed rice biscuits containing different amounts of dandelion complex powder and found that biscuits added with 250–500 mg of dandelion powder significantly improved antioxidant activity and effectively inhibited four pathogenic bacteria such as *Escherichia coli* , with no significant difference in sensory evaluation. Dandelion also can serve as an alternative to capers, being battered and fried into fritters or preserved through cooking in vinegar (Grauso et al. 2019).

### 5.2 Application of Dandelion in Medicine

Dandelion is a perennial herb used both medicinally and as a food, with a long history of clinical application and a wide range of pharmacological effects.

#### 5.2.1 Applications in the Management of Parkinson's Disease (PD) and Gut‐Driven Neurological Disorders

There is a strong association between intestinal microbiota composition and individual differences in the severity and clinical manifestations of PD (Jia et al. 2025). A key translational application of dandelion‐mediated neuroprotection lies in its potential use as a prebiotic or adjuvant therapy targeting the microbiota‐gut‐brain axis. In PD models (e.g., the MPTP‐induced mouse model), the intestinal microbiota shifts toward a pro‐inflammatory profile, characterized by an increase in *Proteobacteria* and a decrease in fatty acid (SCFA)‐producing *Firmicutes* (Jia et al. 2025). Dandelion treatment has been shown to restore the Shannon index (an indicator of alpha diversity) to levels comparable to healthy controls. Recent studies further indicate that dandelion polysaccharides can upregulate tight junction protein expression and enhance intestinal epithelial integrity, thereby reducing LPS leakage into systemic circulation (Li et al. 2024b). By improving microbiota composition and promoting SCFA production, dandelion reduces gut‐derived pro‐inflammatory signals. Thus, dandelion extracts show promising translational potential as functional foods or gut microecology, helping to limit systemic endotoxemia at its source and alleviate PD‐related symptoms (Techaniyom et al. 2024).

#### 5.2.2 Translational Potential in Hepatic Encephalopathy and Metabolic Brain Injury

The liver and brain are metabolically interconnected, and liver dysfunction can result in accumulation of neurotoxins (primarily ammonia) and systemic OS, which contribute to hepatic encephalopathy and amyloid formation (Zhao et al. 2025). As a well‐known hepatoprotective herb, dandelion shows potential for neuroprotection via the liver–brain axis. Dandelion extracts help maintain hepatocyte function and the activity of key enzymes involved in the urea cycle, supporting ammonia detoxification and reducing its neurotoxic effects. In addition, they enhance hepatic antioxidant defenses and inhibit inflammatory signaling pathways such as JAK2, thereby reducing oxidative stress and inflammation in the liver (Salim et al. 2025). These properties suggest that dandelion and its phenolic and terpenoid constituents may serve as promising candidates for the development of “dual liver–brain–targeted” therapeutic agents, particularly for preventing secondary central nervous system damage associated with liver failure or metabolic disorders.

#### 5.2.3 Development of Neuro‐Preventive Agents for Alzheimer's and Neurodegenerative Diseases

Beyond systemic metabolic and microecological regulation, the active components of dandelion are being explored for direct targeted therapeutic applications in neurodegenerative diseases. Chronic activation of microglia is a key pathological hallmark of AD. In LPS‐stimulated BV‐2 microglial cells, pretreatment with dandelion‐derived compounds such as chicoric acid effectively inhibits the production of pro‐inflammatory mediators (e.g., NO and prostaglandin E2) by suppressing the JNK and p38 MAPK pathways, thereby protecting neighboring neurons from inflammatory damage (Tanasa et al. 2025). Recent studies have shown that *Taraxacum officinale* extracts can reverse cognitive impairment symptoms in an aluminum chloride (AlCl_3_)‐induced AD rat model by reducing neuroinflammation and OS. In addition, polyphenols and flavonoids from dandelion leaves retain significant acetylcholinesterase inhibitory activity even after digestion, supporting their potential in cholinesterase‐targeted AD management strategies (Aazza et al. 2023). These findings highlight the potential of dandelion‐derived flavonoids and phenolic acids as lead compounds for the development of next‐generation neuro‐preventive drugs or dietary supplements aimed at slowing the progression of neurodegenerative diseases (Masciulli et al. 2025).

In conclusion, dandelion, as a medicinal plant, holds multiple potential applications in both food and medicine.

## 6 Conclusions and Prospects

As a traditional Chinese medicine with both medicinal and edible properties, dandelion has diverse application potential. This review provides a systematic overview of four active components of dandelion, namely flavonoids, phenolic acids, sterols, and terpenoids, and discusses in‐depth their structures, neuroprotective effects, structure–activity relationships, and practical applications. Structurally, flavonoids possess a basic C6‐C3‐C6 skeleton, while phenolic acids are primarily derivatives of phenylpropanoic acid and benzoic acid. Sterols contain a cyclopentane‐fused polyhydrophthalic acid nucleus, and terpenoids are natural compounds with the general formula (C₅H₈)ₙ. Regarding their neuroprotective functions, these components work synergistically to combat neuroinflammation by regulating cytokines, modulating key inflammatory signaling pathways, and controlling the activation of microglia and astrocytes. Under OS, they counteract damage through strategies such as metal chelation, ROS scavenging, and enhancing endogenous antioxidant defense systems. Their protective effects on neurons are mediated through multiple mechanisms, including reducing A*β* deposition, preventing abnormal tau protein phosphorylation, and optimizing mitochondrial function. In terms of structure–activity relationships, the core skeleton, functional groups, substituents, and unique structural units all play significant roles. Moreover, these active components have demonstrated promising application potential in the food and pharmaceutical industries.

Future research should focus on (1) optimizing the dosage of dandelion active ingredients. For example, studies should optimize the dosage forms and administration routes of dandelion active ingredients to improve their in vivo absorption and inhibition. New methods, such as nanotechnology, controlled release systems, and targeted delivery, must be developed to ensure that the active ingredients of dandelions reach the nervous system and function properly. (2) Conducting cell and animal experiments. For example, exploring the interaction between dandelion active ingredients and nerve cells using animal experiments can better simulate the onset and development of neurological diseases while evaluating the therapeutic effect and safety of the active ingredients. (3) Exploring the combined application of dandelion active ingredients and other treatment methods. We can attempt to combine dandelion active ingredients with traditional medicines or other treatments to enhance therapeutic effects and reduce side effects. By using different treatment methods, we can better manage the complexity of neuroprotection.

## Author Contributions

**Youlin Xue:** methodology, resources, writing – review and editing. **Hongye Li:** conceptualization, writing – original draft. **Bingchan Qu:** methodology, writing – review and editing. **Tiejing Li:** resources, writing – review and editing. **Chongting Guo:** supervision, funding acquisition, writing – review and editing. **Menghan Sun:** visualization, writing – review and editing. **Shan Wang:** methodology, resources, validation, writing – review and editing. **Chong Ning:** writing – review and editing. **Jiasu Wu:** methodology, writing – review and editing. **Chang Tan:** methodology, resources, writing – review and editing.

## Funding

This work was supported by the General Project of the Education Department of Liaoning Province, China, JYTMS20230778, the Science and Technology Plan Joint Program of Liaoning Province, China, 2024‐BSLH‐107, the Enterprise Doctoral Innovation and Entrepreneurship Program of Yingkou, Liaoning Province, China, YKSCJH2024‐017, and the Key Project of the Youth Research Fund of Liaoning University, 2025XJZD003.

## Conflicts of Interest

The authors declare no conflicts of interest.

## Data Availability

The authors have nothing to report.
